# Sorghum and Hemp Responses to Plant Growth-Promoting Microorganism Inoculation in Metal-Contaminated Dredged Sediment: A System-Level Assessment Under Environmentally Relevant Outdoor Pot Conditions

**DOI:** 10.3390/jox16030102

**Published:** 2026-06-02

**Authors:** Marko Šolić, Nina Đukanović, Tamara Apostolović, Jelena Beljin, Irina Jevrosimov, Dragana Tamindžija, Ivana Bajić, Stanko Milić, Tijana Zeremski, Marijana Kragulj Isakovski, Snežana Maletić

**Affiliations:** 1 Faculty of Sciences, University of Novi Sad, Trg Dositeja Obradovića 3, 21000 Novi Sad, Serbia; marko.solic@dh.uns.ac.rs (M.Š.); nina.djukanovic@dh.uns.ac.rs (N.Đ.); tamara.apostolovic@dh.uns.ac.rs (T.A.); irinaj@dh.uns.ac.rs (I.J.); dragana.tamindzija@dh.uns.ac.rs (D.T.); marijana.kragulj@dh.uns.ac.rs (M.K.I.); 2 Institute of Field and Vegetable Crops, National Institute of the Republic of Serbia, Maksima Gorkog 30, 21000 Novi Sad, Serbia; ivana.bajic@ifvcns.ns.ac.rs (I.B.); stanko.milic@ifvcns.ns.ac.rs (S.M.); tijana.zeremski@ifvcns.ns.ac.rs (T.Z.)

**Keywords:** phytoremediation, dredged sediment, plant growth-promoting microorganisms, heavy metals, phytostabilization

## Abstract

Metal-contaminated dredged sediments represent heterogeneous environmental matrices in which remediation responses are frequently constrained by elevated background metal loads and complex geochemical conditions. Within such systems, phytoremediation has been discussed as a nature-based management approach whose outcomes depend on plant biomass, internal metal allocation, and context-dependent interactions between plants and sediment. The present study evaluated whether bacterial and fungal plant growth-promoting microorganisms (PGPMs) were associated with changes in plant metal uptake and internal allocation in *Sorghum bicolor* L. and *Cannabis sativa* L. grown in dredged sediment collected from the Bega Canal. An outdoor pot experiment was conducted under environmentally relevant conditions, including bacterial and fungal inoculation treatments alongside non-inoculated controls, with plant responses to Cr, Ni, Cu, Zn, As, Cd, and Pb characterized using concentration- and mass-based uptake metrics, root–shoot partitioning, and sediment geochemical assessment based on pseudo-total concentrations and BCR sequential extraction fractions. Across treatments, plant responses were largely governed by intrinsic species traits and biomass production, while PGPM-associated effects remained modest and variable. Root-dominated metal retention and limited translocation were evident irrespective of species, consistent with a phytostabilization-type response rather than systematic extraction. Absolute metal uptake accounted for only a minor fraction of total sediment metal pools, underscoring the importance of interpreting concentration-based indices jointly with mass-based metrics when evaluating system-scale responses. Altogether, the findings indicate that under the investigated outdoor dredged sediment pot conditions, PGPM inoculation acts primarily as a context-specific modulator of plant responses rather than a driver of enhanced phytoremediation performance, reflecting the central role of intrinsic plant traits and stabilization-oriented processes in complex sediment systems.

## 1. Introduction

Sediments in inland waterways act as long-term sinks for potentially toxic elements, reflecting the combined effects of historical industrial, municipal, and diffuse anthropogenic inputs. Routine maintenance dredging redistributes these sediments, turning a largely sequestered contamination reservoir into a material that requires active management once removed from the aquatic system. Handling such dredged materials is additionally complicated by their pronounced physicochemical heterogeneity, mixed contamination profiles, and the disturbance of geochemical equilibria upon excavation. These challenges are further amplified by the large volumes of material generated during maintenance dredging, the costs associated with transport, treatment, and disposal, and regulatory constraints governing the safe reuse or final management of contaminated sediments. Consequently, remediation or beneficial reuse of dredged sediments is constrained by both environmental risk concerns and practical limitations arising from their complex composition [1,2,3,4].

Such complexity has direct implications for remediation performance, as the physicochemical heterogeneity of dredged sediments gives rise to spatially variable metal pools and constrains the predictability of treatment outcomes. Within these matrices, metals are distributed among multiple solid phases and binding environments, leading to uneven availability and heterogeneous response patterns even under nominally identical treatment conditions. Accordingly, remediation responses documented in simplified or highly controlled experimental systems may not translate directly to complex dredged sediments, where context-dependent behavior often plays a central role [5,6,7,8].

Metal-contaminated soils and sediments may be managed using conventional physical, chemical, and thermal treatment methods, such as excavation and replacement, capping or containment, soil washing, solidification/stabilization, immobilization, electrokinetic remediation, and vitrification. Ex situ approaches can provide relatively rapid contaminant removal or risk reduction, particularly in localized or highly contaminated materials, but they may also require intensive material handling, substantial energy or reagent inputs, and high costs, while potentially altering the physical, chemical, or biological properties of the treated substrate. In situ approaches can reduce excavation and transport requirements, although their effectiveness may be constrained by metal speciation, treatment duration, and the long-term stability of the treated material. For dredged sediments, method selection is further complicated by large material volumes and by the need to balance contaminant reduction with disposal, beneficial reuse, and long-term environmental safety. Accordingly, lower-input biological approaches remain relevant as complementary alternatives, particularly where risk reduction, stabilization, ecological compatibility, and resource-efficient management are prioritized over rapid contaminant removal [4,6,9].

In this context, phytoremediation has been proposed as a nature-based approach for managing metal-contaminated soils and sediments, relying on plant–substrate interactions under prevailing environmental conditions rather than intensive physical or chemical interventions. Depending on system properties and management objectives, phytoremediation is commonly discussed in terms of phytoextraction and phytostabilization, which differ in their emphasis on contaminant removal versus immobilization within the substrate. Across complex matrices, phytoremediation outcomes have, however, been described to depend less on tissue concentration metrics alone and more on plant biomass production, root retention, and internal metal partitioning. Accordingly, in substrates such as dredged sediments, phytoremediation has been discussed primarily in terms of modulation and stabilization of metal behavior rather than consistent removal from the system [10,11,12,13,14].

Against this background, plant growth-promoting microorganisms (PGPMs), including both bacterial and fungal inoculants, have been explored as potential modifiers of plant–metal interactions in contaminated soils and sediments. At a more conceptual level, such microorganisms have been discussed in relation to their influence on rhizosphere-mediated processes, including nutrient availability, root development, and metal availability and partitioning. However, reported PGPM effects on metal uptake and internal plant metal handling have been highly variable across studies, with responses shown to depend on substrate properties, plant species, and cultivation conditions. Together, this variability highlights the importance of evaluating PGPM responses within realistic systems that reflect the complexity of contaminated substrates [15,16,17,18,19].

The present study was designed to evaluate whether bacterial and fungal PGPM inoculation was associated with changes in metal uptake and internal metal allocation in plants grown in contaminated dredged sediment under environmentally relevant conditions. To address this goal, the study was conducted as an outdoor pot experiment, with *Sorghum bicolor* L. and *Cannabis sativa* L. selected as model plant species. Plant biomass production and root–shoot metal partitioning were examined to characterize system-level patterns in plant metal handling under these conditions.

To capture system-level responses, the study incorporated concentration-based and mass-based metrics of plant metal uptake with indicators of internal metal allocation between roots and shoots. Sediment metal pools were quantified to provide environmental context for plant responses, rather than to serve as direct predictors of metal uptake or internal partitioning. By using real contaminated dredged sediment and a system-level evaluation of plant metal handling, the study builds on previous PGPM phytoremediation research implemented under simplified or highly controlled conditions. The study was not designed to infer mechanistic causality or to demonstrate systematic enhancement, but rather to assess context-dependent responses at the system level under environmentally relevant outdoor pot conditions.

## 2. Materials and Methods

### 2.1. Sediment Origin and Characterization

The sediment used for this study originated from a section of the Bega Canal in Serbia, which constitutes part of the Danube–Tisa–Danube (DTD) hydrosystem connecting the Danube and Tisa rivers. This canal network has been subjected to long-term inputs from industrial effluents, municipal wastewater, and diffuse agricultural runoff, resulting in the progressive accumulation of heavy metals in bottom sediments [20].

For the present study, dredged sediment was collected from a confined disposal area containing material removed during previous maintenance dredging of the Bega Canal. The disposal area is located near Srpski Itebej, close to the Serbian–Romanian border, extending approximately between 45°34′48.91″ N, 20°45′28.01″ E and 45°34′50.03″ N, 20°45′30.86″ E. Approximately 500 kg of bulk sediment material was collected from ten points across the disposal site using an excavator, combined to obtain a representative bulk composite sample, transported by truck to the experimental facility under ambient conditions, and mechanically homogenized prior to use. The homogenized sediment was employed as the growth substrate for all treatments.

The physicochemical properties and contamination status of the dredged sediment have been previously described in detail [21] and provided the foundation for the present pot experiment. Baseline sediment characteristics and heavy metal concentrations were determined again prior to the pot experiment to confirm its suitability and to obtain an independent dataset for the current study.

As indicated by the values shown in Table 1, the sediment was alkaline, sandy loam in texture, contained moderate organic matter and measurable nutrient contents, and was characterized by a relatively high cation exchange capacity and non-saline conditions.

### 2.2. Pot Experiment Design

The pot experiment was conducted outdoors under natural environmental conditions in Rimski Šančevi, Serbia, employing dredged sediment as the growth substrate and two test species, forage sorghum (*Sorghum bicolor* L.) and hemp (*Cannabis sativa* L.). Certified seeds of *S. bicolor* cv. NS Džin and *C. sativa* cv. HELENA were obtained from the Institute of Field and Vegetable Crops, Novi Sad, Serbia, with detailed seed-lot quality parameters provided in Table S1. These species were selected as fast-growing, high-biomass annual crops with documented tolerance to metal-contaminated conditions and relevance for phytoremediation and phytomanagement of contaminated soils and sediments, providing two model plant systems for assessing PGPM-associated responses under the same dredged sediment conditions [21,22,23,24].

Each pot was loaded with 5 kg of air-dried and homogenized sediment that had been prepared immediately prior to the experiment. Five seeds were sown per pot, and upon germination, the seedlings were thinned to retain a single plant per pot. The experimental design encompassed three treatments for each species—control (no inoculant; S or H), bacterial inoculation (S/B or H/B), and fungal inoculation (S/F or H/F)—with each treatment established in triplicate. Both plant-growth-promoting inoculants were supplied as liquid commercial formulations, including a bacterial PGPR-based product (GROUNDFIX) and a fungal inoculant (MYCOFRIEND), and were applied once just before sowing. The inoculants were not experimentally formulated or optimized within this study; instead, the manufacturer-specified field-equivalent quantities (4 and 0.5 L/ha, respectively) were used. These quantities were diluted in distilled water to obtain an adequate working volume and uniformly sprayed over the sediment prior to pot filling. The cultivation period lasted 10 weeks, from 25 April 2024 to 3 July 2024, and was selected based on plant development during the experiment and previous experience from comparable contaminated-sediment cultivation studies [21,25].

For the cultivation period, key meteorological parameters were obtained from Meteostat [26], using data from the Novi Sad–Rimski Šančevi meteorological station, the nearest measuring station to the experimental site, and are summarized in Table S2. Because the experiment was conducted outdoors, air temperature, precipitation, and light exposure reflected naturally occurring meteorological conditions rather than controlled laboratory or greenhouse settings. During the 10-week cultivation period, the average air temperature was 20.9 °C, with daily mean temperatures ranging from 9.5 to 29.3° C. Cumulative precipitation amounted to 163.7 mm, with precipitation recorded on 28 days. The average daylight and dark periods were approximately 15 h and 9 h, respectively, with an average sunshine duration of approximately 480 min/day. Pots were irrigated with tap water as needed, according to plant developmental stage and prevailing outdoor conditions, together with visual assessment of substrate moisture. Irrigation was applied carefully to maintain plant growth while avoiding excessive wetting and visible leaching from the pots; however, substrate moisture was not continuously instrument-monitored, and leachate was not collected or chemically analysed.

At harvest, each plant was removed from its corresponding pot and processed as an individual biological replicate. Shoots and roots were separated and thoroughly rinsed with distilled water to remove adhering particles. The plant material was then dried at 40 °C, weighed, and subsequently ground to a fine powder prior to chemical analysis.

Prior to sowing, sediment allocated for each treatment was sampled to obtain a composite pre-harvest sample, derived by combining representative subsamples from the homogenized sediment batch. At harvest, sediment from the three replicate pots within each treatment was collected and pooled to generate one post-harvest composite sample per treatment. This approach was used to obtain an integrated treatment-level sediment sample in a matrix characterized by microscale heterogeneity and to provide a geochemical context for plant responses. Accordingly, post-harvest pseudo-total concentrations and BCR fractions were interpreted descriptively as treatment-level sediment data, rather than as explanatory factors for differences observed among plant biological replicates. All pre- and post-harvest sediment samples were air-dried, ground, and sieved prior to chemical analysis.

### 2.3. Assessment of Phytoremediation Efficiency

Phytoremediation performance was evaluated using standard soil-to-plant transfer indices. The bioaccumulation factor (BAF) was calculated separately for roots and shoots as the ratio of metal concentration in plant tissue (*C*_plant_) to the corresponding metal concentration in sediment (*C*_sed_). The translocation factor (TF) was determined as the ratio of metal concentration in shoots (*C*_shoot_) to that in roots (*C*_root_). The calculations of BAF and TF were performed according to Equations (1) and (2), respectively [27,28].

(1) $$BAF=C_{plant\;(shoot\;or\;root)}/C_{sed}$$

(2) $$TF=C_{shoot}/C_{root}$$

### 2.4. Chemical and Analytical Methods

Physicochemical properties of the sediment were assessed according to the protocols described below: pH was measured potentiometrically in a 1:5 (*w*/*v*) sediment-to-deionized water suspension after 60 min of equilibration, with a glass electrode [29]. Electrical conductivity was determined in a 1:5 sediment-to-deionized water extract following a 30 min agitation period [30]. Organic matter content was quantified by the loss-on-ignition method subsequent to combustion at 550 °C [31]. Total nitrogen was determined using the Kjeldahl method, consisting of sulfuric acid digestion followed by distillation and titration [32]. Total phosphorus was quantified after extraction with ammonium lactate, with subsequent colorimetric detection using the molybdenum blue procedure and measurement by UV–Vis spectrophotometry (Shimadzu UV-1800, Shimadzu Corp., Kyoto, Japan), according to an internal laboratory protocol. Total potassium was determined after microwave-assisted digestion (Start E, Milestone, Sorisole, Italy), with subsequent measurement by flame emission spectrophotometry employing an atomic absorption spectrometer (iCE 300 series, Thermo Fisher Scientific, Cambridge, UK), as specified in Method 3500-K D [33]. Cation exchange capacity was assessed by ammonium acetate extraction (1 M NH_4_OAc, pH 7.0) following a method modified from van Reeuwijk [34], calculated based upon the sum of exchangeable base cations and expressed as cmol_(c)_/kg dry weight. Exchangeable Ca^2+^ and Mg^2+^ were quantified by graphite furnace atomic absorption spectrometry following Method 7010 [35], while Na^+^ and K^+^ were determined by flame emission spectrophotometry in accordance with Methods 3500-Na B and 3500-K D [33], respectively. Particle size distribution was evaluated by dry sieving for the sand fraction and sedimentation analysis for the silt and clay fractions [36].

Pseudo-total metal concentrations in sediment and plant samples were determined after microwave-assisted acid digestion as specified in Method 3051A [37]. The resulting digests were analyzed by atomic absorption spectrometry employing graphite furnace or flame modes, as appropriate (refer to instrumentation details above), in accordance with Methods 7010 and 7000B, respectively [35,38]. Element-specific AAS conditions and validated method-performance parameters are provided in Supplementary Table S3. The measured elements included Cr, Ni, Cu, Zn, As, Cd, and Pb. Although As is chemically classified as a metalloid, it is commonly discussed together with heavy metals in environmental contamination studies; therefore, for consistency and readability, the analysed elements are collectively referred to as “heavy metals” throughout the manuscript.

Operationally defined metal fractions in sediment were obtained by applying the BCR sequential extraction procedure following the method described in [39]. Metal concentrations in the extracts were quantified by atomic absorption spectrometry under the same instrumental conditions.

### 2.5. Data Analysis

**Figure 1:**
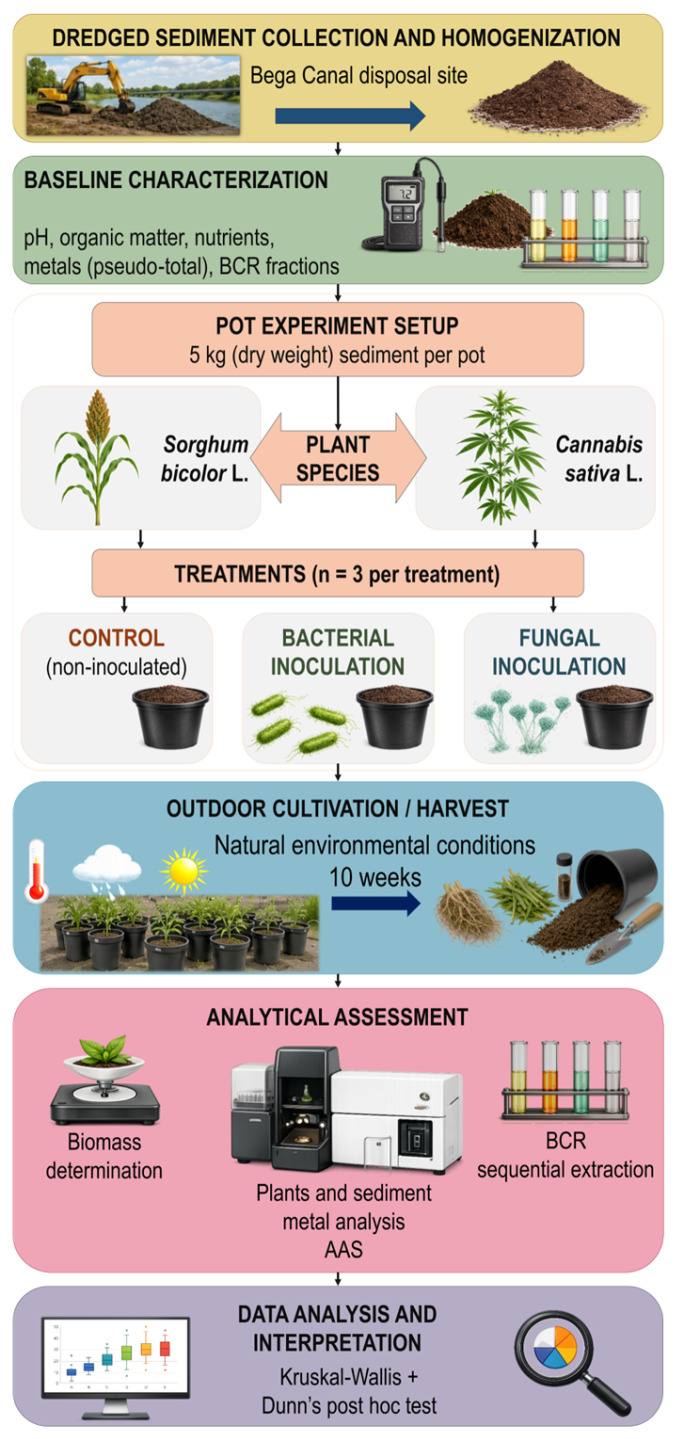
Schematic overview of the experimental workflow and methodological scope of the study.

Statistical analyses were performed using TIBCO Statistica, version 14.1 (TIBCO Software Inc., Palo Alto, CA, USA). All statistical procedures were performed exclusively on plant datasets comprising three biologically independent replicates per treatment. Plant data were summarised as means ± standard deviations, and individual replicate values were plotted to visualise biological variability within treatments. Non-parametric statistical methods were used as the small number of biological replicates did not permit reliable assessment of normality, and environmental datasets typically deviate from parametric assumptions. Within each plant species, treatment differences (S vs. S/B vs. S/F and H vs. H/B vs. H/F) were evaluated by means of the Kruskal–Wallis test, followed by Dunn’s post hoc comparisons with Bonferroni correction for multiple testing. Direct statistical comparisons between sorghum and hemp were not performed because the two species represent distinct plant systems rather than alternative treatment levels; therefore, cross-species differences were summarised descriptively. Statistical significance was defined as *p* < 0.05 [40,41,42]. Figures were constructed using OriginPro 8.5 (OriginLab Corporation, Northampton, MA, USA). The overall experimental workflow is summarized in Figure 1.

## 3. Results

### 3.1. Sediment Heavy Metal Fractions Before and After Harvest (Pseudo-Total and BCR)

#### 3.1.1. Pseudo-Total Concentrations

**Figure 2:**
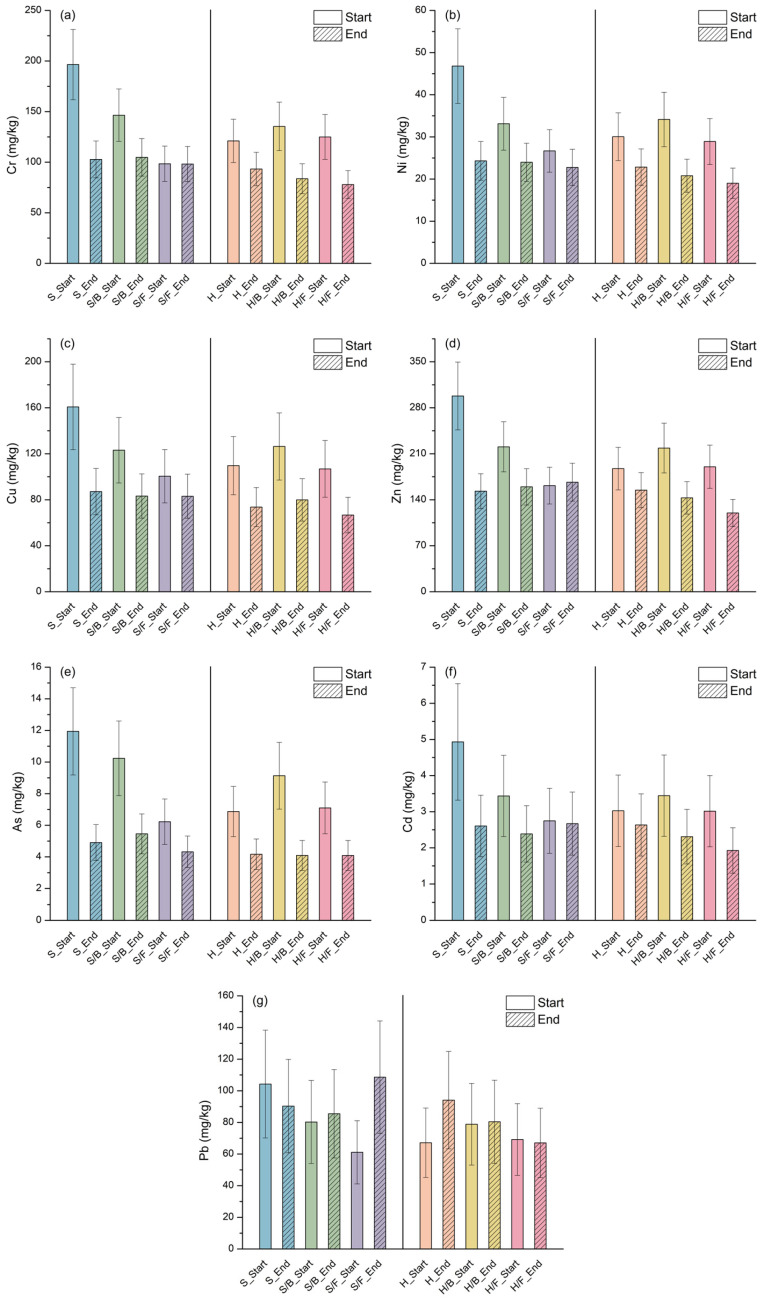
Pseudo-total concentrations of (**a**) Cr, (**b**) Ni, (**c**) Cu, (**d**) Zn, (**e**) As, (**f**) Cd, and (**g**) Pb in sediment before sowing and after harvest across all investigated treatments. Sediment data were obtained from composite samples, each analysed once. Error bars indicate analytical measurement uncertainty.

The pseudo-total concentrations of the selected heavy metals in the sediment, measured before sowing and after harvest across all investigated treatments, are presented in Figure 2. At the start of the experiment, the relative abundance followed Zn > Cr > Cu > Pb > Ni > As > Cd, with median concentrations of approximately 204.5, 130.2, 116.4, 74.0, 31.6, 8.1, and 3.2 mg/kg, respectively. The variations in baseline concentrations reflect the inherent heterogeneity of the contaminated sediment, despite homogenization prior to application.

To place these concentrations in a regulatory context, they were compared with Dutch soil-quality thresholds under the current Regeling bodemkwaliteit 2022 framework, using the Agriculture/Nature quality values for land soil and dredged material applied on land, as reported in Annex B, Table 1 of the regulation [43]. Dutch soil-quality thresholds are frequently used as reference values in environmental assessments, particularly in the absence of harmonised EU-wide soil quality standards [44]. Based on this comparison, Cr, Cu, Zn, Cd, and Pb exceeded the Agriculture/Nature threshold values across all starting composites, whereas Ni exceeded the threshold in only one composite and As remained below the corresponding threshold in all baseline samples. Despite the lower exceedance frequency for Ni and the absence of exceedance for As, both elements were retained for further assessment given the documented contamination history of the Bega Canal system and their potential ecological relevance in dredged and recultivated sediments [20].

Following harvest, pseudo-total concentrations generally declined for Cr, Ni, Cu, As, and Cd, while Pb, in multiple cases, and Zn, in a single case, exhibited increases, indicating heterogeneous overall behaviour among the elements. Observed declines ranged from minimal (<1%) to marked (~59%), whereas increases, most notably in Pb, reached ~78%. Considering only the heavy metals that consistently declined across treatments, percentage changes ranged from ~0–48% for Cr, ~15–48% for Ni, ~17–46% for Cu, ~31–59% for As and ~3–47% for Cd. Regarding Zn and Pb, the ranges were as follows: ~−3% (gain) to ~49% for Zn and ~−78% (gain) to ~13% for Pb.

At the treatment level, in sorghum systems, percentage changes were generally lower under both bacterial and fungal inoculation compared with the control. Notably, fungal applications consistently showed the smallest relative reductions (S/F < S/B < S). In hemp systems, however, percentage changes tended to be higher compared with the control, lacking a consistent distinction between the two (H < H/B and H < H/F). Comparing the tested plant systems, sorghum showed greater percentage reductions under control conditions (H < S), whereas following inoculation, the response pattern shifted in the opposite direction (S/B, S/F < H/B, H/F), reflecting the trends previously noted.

These observed percentage trends should be interpreted with caution, as analytical uncertainties (up to ~33% for Cd and Pb) may be compounded when comparing initial and final values, potentially overshadowing apparent differences. Additionally, variations in initial metal concentrations between treatments—such as higher starting levels in the S—could influence the apparent magnitude of change, particularly when expressed solely on a relative basis rather than in absolute terms. Accordingly, both recorded decreases and treatment-specific cases with minimal start–end change were interpreted as descriptive trends in treatment-level composite sediment data rather than as direct evidence of specific inoculant-related effects.

To complement the percentage trends, absolute changes in total metal mass per pot were assessed, providing a clearer indication of the actual magnitude of metal depletion across treatments. Among the analysed metals, Zn, Cr, and Cu exhibited the largest absolute losses, with Zn ranging from a slight gain (~26 mg) to a maximum loss of ~724 mg per pot, Cr from near-zero change to ~469 mg, and Cu from ~87 to ~368 mg, depending on treatment. By comparison, Ni showed more moderate losses, ranging from ~20 to ~112 mg, while As and Cd exhibited only minor absolute changes of ~9 to ~35 mg and ~0.4 to ~12 mg, respectively. In contrast, Pb demonstrated net gains in most treatments, reaching up to ~238 mg, opposing the overall depletion trends observed for the other metals. The corresponding within-treatment trends in total metal mass per pot, derived from the differences between pre- and post-harvest concentrations, are presented in Table 2.

#### 3.1.2. BCR Fractions

**Figure 3:**
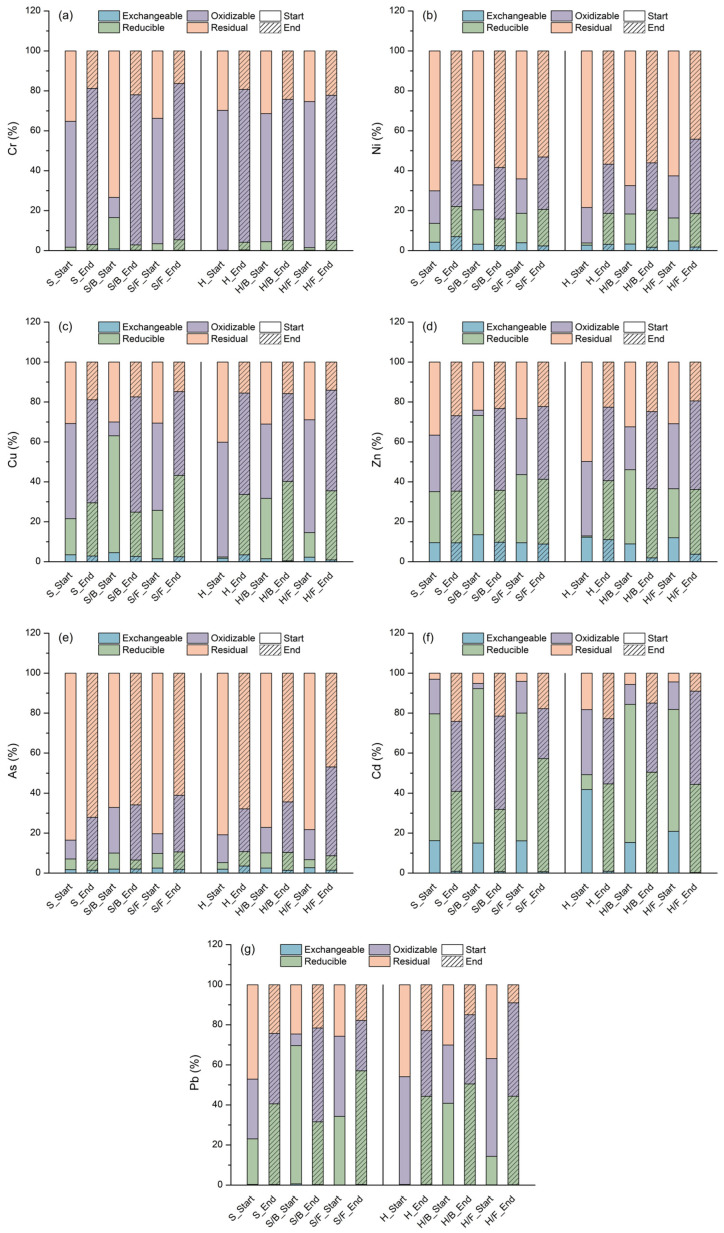
Fractional distribution of (**a**) Cr, (**b**) Ni, (**c**) Cu, (**d**) Zn, (**e**) As, (**f**) Cd, and (**g**) Pb among the operationally defined BCR fractions in sediment before sowing and after harvest across all investigated treatments. Sediment data were obtained from composite samples, each analysed once.

The distribution of the selected heavy metals among the operationally defined BCR fractions before sowing and after harvest across all investigated treatments is presented in Figure 3, providing insight into changes in metal partitioning and potential mobility beyond total concentrations. Examination of the baseline data reveals distinct pre-sowing variations within individual fractions, likely showing microscale heterogeneity in the contaminated sediment and the uneven distribution of reactive mineral and organic phases. For ease of comparison, the metals were grouped according to their dominant baseline association within the BCR fractions. Of these, Cr and Ni were mainly retained in low-mobility fractions—oxidisable and residual—which together comprised ~83–100% and ~80–96% of their total content, respectively. A comparable pattern was observed for Cu, with ~68–98% in these fractions. In contrast, Zn and Pb exhibited somewhat broader and more balanced distributions across the reducible, oxidisable, and residual phases. However, the low-mobility fractions still constituted the largest share, comprising ~54–87% and ~59–100%, respectively. Finally, As and Cd represented the extremes of immobility and lability, with the residual fraction containing ~67–84% of As, whereas the combined exchangeable and reducible fractions comprised ~49–92% of Cd at baseline. Notably, the reported ranges were derived excluding the S/B treatment for Cu, Zn, and Pb, as this variant—similar to the H—showed irregular phase distributions that deviated from the general baseline pattern. Overall, by considering the exchangeable and reducible fractions combined—constituting the mobile metal pool—the relative potential mobility followed the approximate order Cd > Zn > Pb > Cu > Ni > As > Cr, with only marginal variations among the baseline sediment samples.

After harvest, observable shifts were recorded among the BCR fractions. For most elements—particularly Cr, Ni, Cu, Zn, As, and Pb—the residual fraction decreased, accompanied by moderate increases or internal redistributions within the reducible and oxidisable phases. The magnitude of these changes generally remained below 30%, with occasional higher shifts identified in specific treatments, most notably in the S/B and H variants. In contrast, the exchangeable fraction remained essentially unchanged, contributing less than 5% in most cases. A distinct pattern emerged for Cd, characterised by a pronounced reduction in the combined share of the exchangeable and reducible fractions, ranging from ~23–39%, with the S/B and H once again differing markedly (~60% and ~5%, respectively). Despite these redistributions, the relative sequence within the mobile metal pool remained largely similar to the baseline, following the approximate order Cd ≈ Pb > Zn > Cu > Ni > As > Cr, with only minor treatment-related variations.

At the treatment level, in sorghum systems, bacterial inoculation generally reduced the mobile metal pool relative to the control, whereas fungal application increased it for all elements other than Ni and Zn (S/B < S < S/F; Δ-direction). In hemp systems, both inoculants reduced the mobile pool relative to the control, with bacterial treatment showing the greater decline, and Cd being the only exception (H/B < H/F < H). Comparing the tested plant systems, hemp exhibited greater increases in the mobile pool across the corresponding treatments, apart from Cd under fungal inoculation (S < H).

As previously noted for the pseudo-total data, these BCR-based trends should be interpreted with caution, as various fractional shifts fall within the range of analytical uncertainty and may not fully reflect true changes. In addition, the irregular phase distributions observed in the S/B and H variants could influence the apparent treatment effects, particularly when comparing relative rather than absolute differences.

### 3.2. Plant Biomass, Uptake, and Tissue Partitioning of Heavy Metals

#### 3.2.1. Biomass

**Figure 4:**
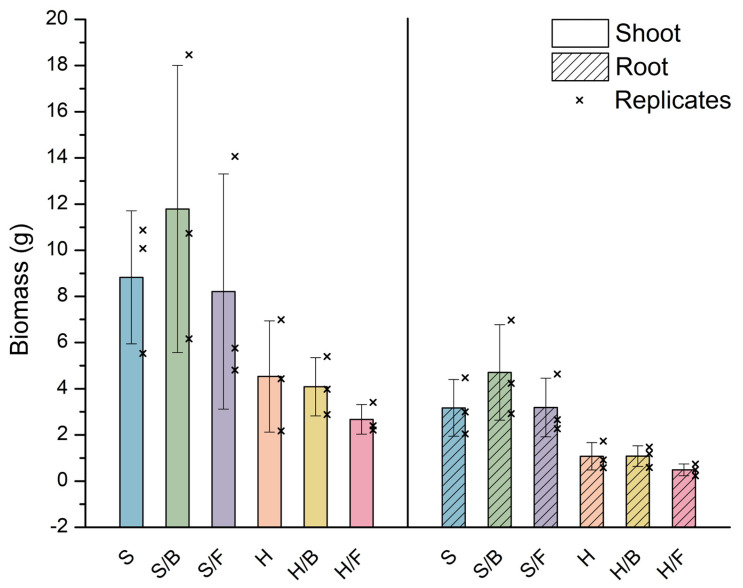
Dry biomass of shoots and roots for sorghum and hemp across all investigated treatments. Bars represent mean values (n = 3) ± SD, with individual replicates shown as cross marks.

The dry biomass values for shoots and roots across all investigated treatments are presented in Figure 4. As indicated, shoot biomass consistently exceeded root biomass in both plant species. In sorghum, shoot dry biomass ranged from ~4.8–18.5 g/pot and root biomass from ~2.0–7.0 g/pot, whereas hemp produced ~2.2–7.0 g/pot in shoots and ~0.2–1.7 g/pot in roots. Mean biomass patterns further suggested that sorghum followed the order S/B > S ≈ S/F for both shoots and roots, while in hemp shoot, biomass ranked as H > H/B > H/F and root biomass as H ≈ H/B > H/F. Across corresponding treatments, sorghum uniformly produced greater shoot and root biomass than hemp (S > H). However, none of these differences were statistically significant (all *p* ≥ 0.19).

#### 3.2.2. Metal Concentrations

**Figure 5:**
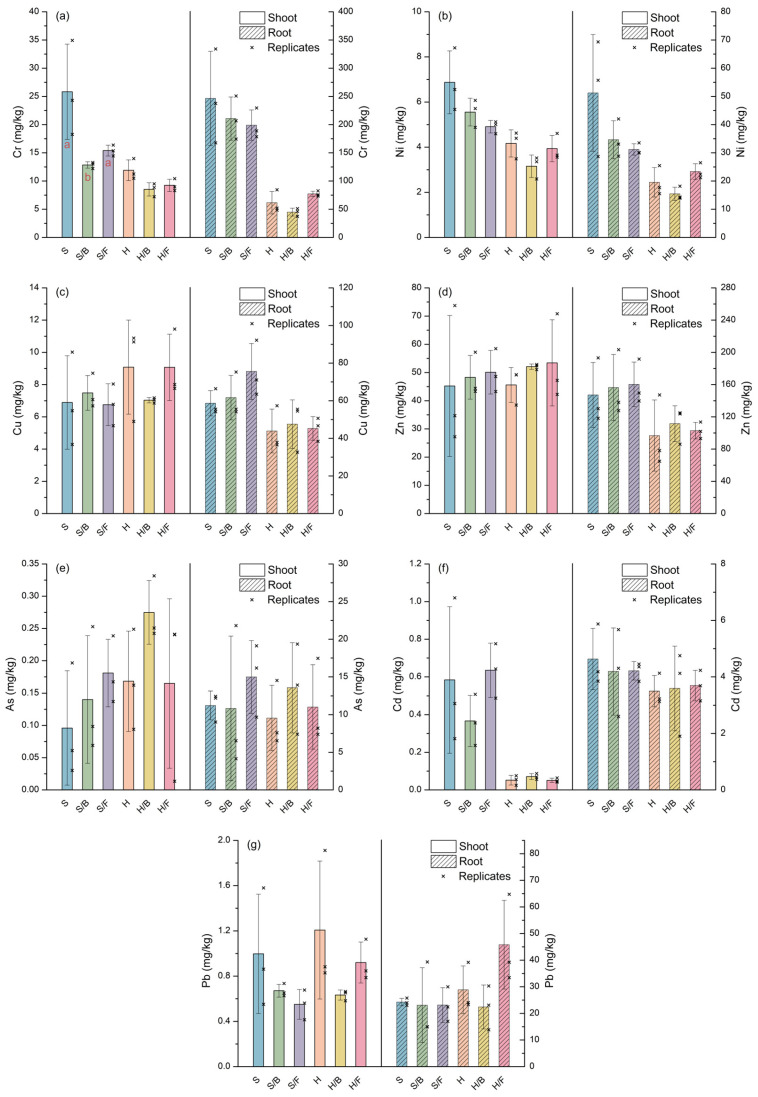
Concentrations of (**a**) Cr, (**b**) Ni, (**c**) Cu, (**d**) Zn, (**e**) As, (**f**) Cd, and (**g**) Pb in shoots and roots of sorghum and hemp across all investigated treatments. Shoot and root concentrations are plotted against the left and right y-axes, respectively. Bars represent mean values (n = 3) ± SD, with individual replicates shown as cross marks. Different letters denote significant differences among treatments within each plant species and tissue (*p* < 0.05).

The concentrations of the selected heavy metals in shoots and roots for all investigated treatments are presented in Figure 5. Overall, metal concentrations encompassed wide ranges among elements and tissues, with roots showing higher concentrations than shoots across the investigated treatments. Across elements, root concentrations ranged from low tens to over 200 mg/kg, with Cr, Zn, and Cu exhibiting the highest values, whereas shoot concentrations were markedly lower, with Zn being the dominant element but still remaining below ~60 mg/kg.

At the treatment level, sorghum roots showed declining Cr and Ni concentrations from S to the inoculated variants, reaching the lowest values in S/F. Conversely, elevated concentrations of Cu and As were observed in S/F compared with S and S/B, while Zn, Cd, and Pb differed by ≤10% across variants. In shoots, Cr, Ni, and Pb were highest in S and lower in the inoculated variants, with Ni and Pb declining from S to S/B and S/F, while Cu and Zn varied only marginally (≤10%). Shoot As was lowest in S and increased toward S/F, whereas Cd showed an opposite trend, with the lowest values in S/B and the highest in S/F. Despite the described treatment-level shifts, none of the differences in metal concentrations among S, S/B, and S/F were statistically significant for roots (all *p* ≥ 0.25), whereas in shoots, only Cr showed a significant treatment effect (*p* = 0.027), driven by higher concentrations in S compared with S/B (post hoc *p* = 0.022). All other pairwise comparisons were non-significant (all *p* ≥ 0.11).

In hemp roots, Cr, Ni, and Pb were lowest in H/B and highest in H/F, with H showing intermediate values. A similar pattern occurred for Zn and As, with elevated levels in H/B relative to H and H/F; however, the increase was slight for Zn but more pronounced for As. Marginal variation was found for Cu and Cd across variants. In shoots, Cr, Ni, and Pb were highest in H and declined under inoculation, with intermediate levels in H/F and the lowest in H/B. A comparable trend was observed for Cu, with H and H/F exceeding H/B. For Zn, concentrations were higher in H/B and H/F than in H, while the difference between the two inoculated treatments remained comparatively modest. In contrast, As and Cd showed the opposite trend, with the highest levels in H/B and lower concentrations in H and H/F. Despite these treatment-level differences, none of the variations in metal concentrations among H, H/B, and H/F were statistically significant for either roots (all *p* ≥ 0.079) or shoots (all *p* ≥ 0.061).

Across corresponding treatments, root concentrations tended to be higher in sorghum than in hemp (S > H), whereas shoot concentrations were broadly comparable between the two species (S ≈ H).

#### 3.2.3. Metal Uptake

**Figure 6:**
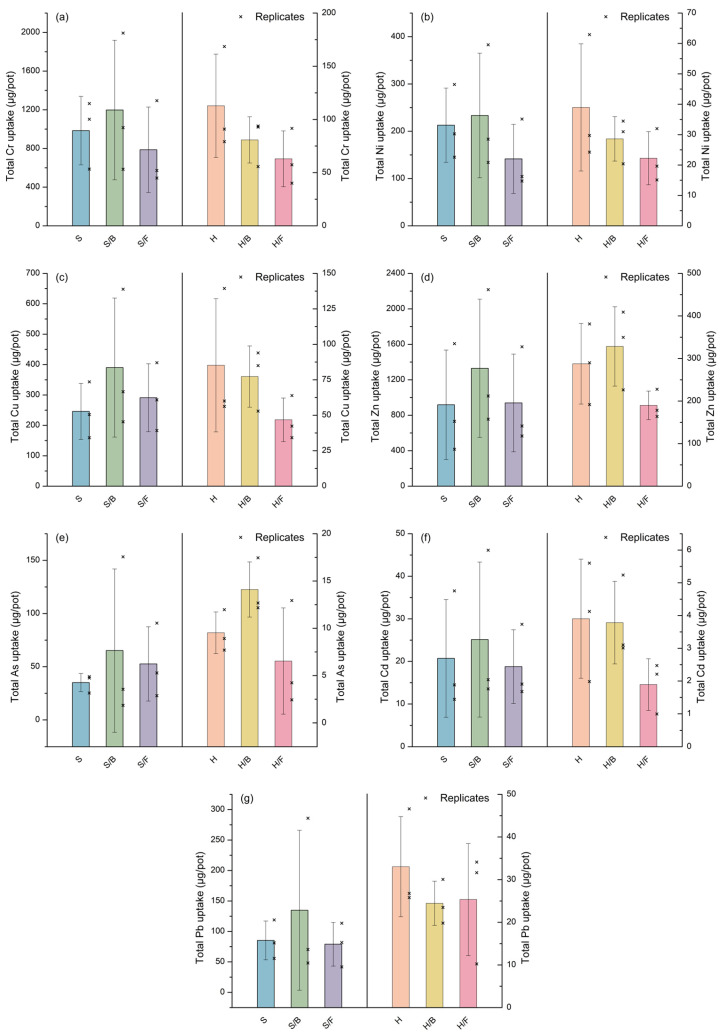
Total uptake of (**a**) Cr, (**b**) Ni, (**c**) Cu, (**d**) Zn, (**e**) As, (**f**) Cd, and (**g**) Pb per pot across all investigated treatments. Sorghum and hemp treatments are plotted against the left and right y-axes, respectively. Bars represent mean values (n = 3) ± SD, with individual replicates shown as cross marks.

In addition to tissue concentrations, total metal uptake per pot was derived for all investigated treatments (Figure 6). Across metals and variants, total uptake spanned broad ranges, from only a few micrograms to over 1 mg, with Zn and Cr exhibiting the highest overall amounts, followed by Cu and Ni, whereas Pb, As, and Cd remained comparatively low.

At the treatment level, sorghum demonstrated similar patterns for Cr, Ni, Cd, and Pb, with total uptake increasing from S to S/B and declining toward S/F. For Cu, Zn, and As, uptake was likewise highest in S/B, with S/F exceeding or approximating S in an element-dependent manner. Despite these treatment-level differences, none of the variations in total uptake among S, S/B, and S/F were statistically significant for any metal (all *p* ≥ 0.43).

In hemp, Cr, Ni, and Cu showed declining total uptake from H to the inoculated variants, with the lowest values consistently observed in H/F. For Zn and As, uptake was highest in H/B, with H exhibiting intermediate amounts and H/F remaining lowest. For Cd, uptake was similar in H and H/B and declined toward H/F, while for Pb the highest values occurred in H, with both inoculated variants showing lower and closely comparable amounts. Despite these treatment-level shifts, none of the differences in total uptake among H, H/B, and H/F were statistically significant for any metal (all *p* ≥ 0.18).

Across corresponding treatments, total metal uptake was consistently higher in sorghum than in hemp for all analysed elements (S > H).

#### 3.2.4. Bioaccumulation Factors

To characterise sediment-to-plant metal accumulation, BAF values were derived for roots and shoots across all treatments and summarised in Table 3. The resulting BAFs spanned wide ranges among elements and tissues, with roots (~0.2–3.5) systematically showing higher magnitudes than shoots (~0.005–0.45), indicating greater relative sediment-to-tissue accumulation in roots than in shoots. Within the metals, As displayed the highest root BAFs, reaching up to ~3.5. Values for Cr and Ni fell within an intermediate-to-high range (~0.5–2.4), whereas Cd remained within a moderate interval (~1.3–1.9). In comparison, Cu and Zn demonstrated lower BAFs (~0.6–1.0), and Pb consistently presented the lowest values (<0.7). Shoot BAFs were uniformly low, with only Zn showing modestly higher levels compared to the other metals, which stayed within narrow ranges.

At the treatment level, root BAFs in sorghum declined for a number of metals, with Cr and Ni exhibiting lower values under both inoculated variants compared to S, while differences between S/B and S/F remained marginal (≤10%). For both Cd and Pb, values displayed minimal variation between S and S/B, followed by a reduction in S/F. In contrast, Cu showed increasing values across the treatments, rising slightly in S/B and more clearly in S/F. A distinct pattern was observed for As, with BAFs decreasing from S to S/B and then increasing markedly in S/F, exceeding those in both preceding treatments. Regarding Zn, BAFs recorded only minor variation across S, S/B, and S/F. Despite these treatment-level differences, none of the variations in root BAFs among S, S/B, and S/F were statistically significant (all *p* ≥ 0.11).

In hemp, root BAFs for Cr and Ni declined from H to H/B and then increased in H/F. Similarly, Pb exhibited a comparable directional pattern, with a slight decrease in H/B and a marked increase in H/F. Both Zn and Cd showed consistent increases across the hemp treatments, with higher BAFs in H/B than in H and further rises in H/F. A distinct sequence of changes was observed for As, with BAFs increasing from H to H/B and declining in H/F, remaining higher than the control but lower than the bacterial treatment. For Cu, BAFs were largely unchanged between H and H/B and then increased modestly in H/F. Despite these treatment-level differences, none of the variations in root BAFs among H, H/B, and H/F were statistically significant (all *p* ≥ 0.061).

Across corresponding treatments, sorghum generally recorded higher root BAFs than hemp for several metals, most notably Cr, Ni, Cu, and Zn. By comparison, Pb tended to be higher in hemp, although the difference in the bacterial variant was marginal. A mixed response was observed for Cd, with sorghum showing higher BAFs in both the control and bacterial treatments, whereas hemp recorded higher values in the fungal treatment. For As, root BAFs were similar between the species in the control, higher in hemp under bacterial inoculation, and shifted toward sorghum in the fungal treatment.

#### 3.2.5. Root–Shoot Partitioning

**Figure 7:**
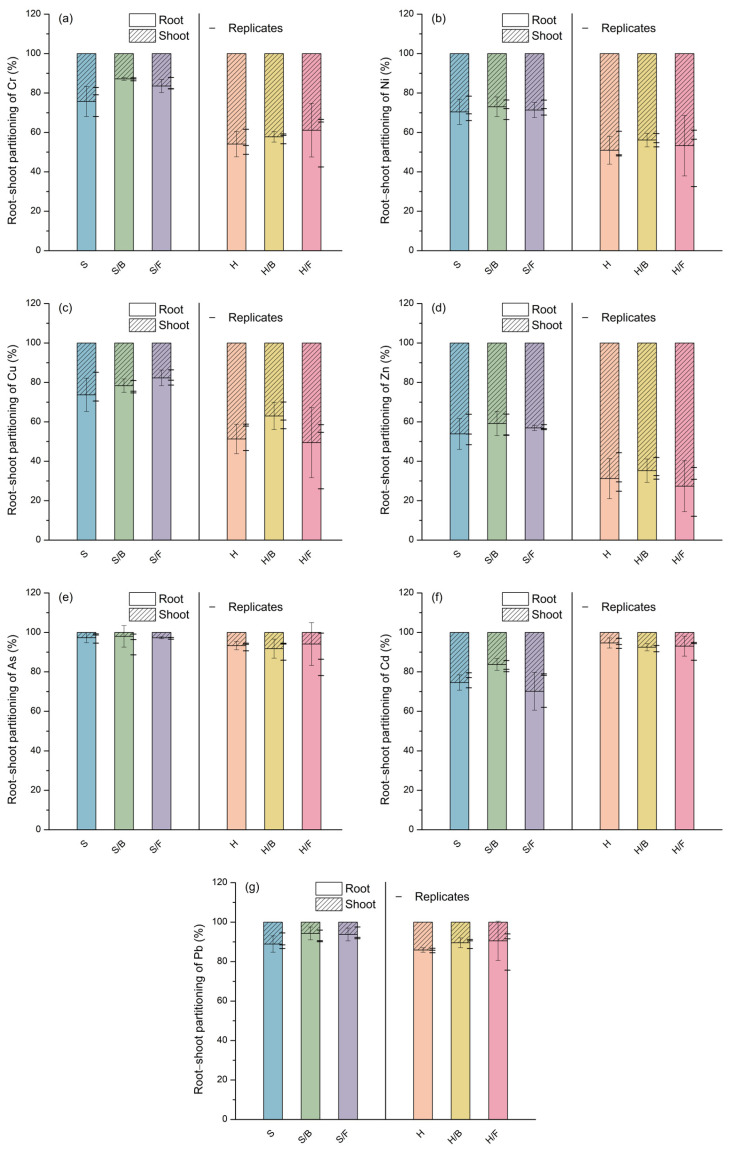
Root–shoot partitioning of (**a**) Cr, (**b**) Ni, (**c**) Cu, (**d**) Zn, (**e**) As, (**f**) Cd, and (**g**) Pb expressed as percentage contributions to total uptake across all investigated treatments. Bars represent mean values (n = 3), with individual replicates shown as horizontal tick marks.

To further characterise metal allocation within the plants from a mass-based perspective, root–shoot partitioning was assessed based on the relative contributions of roots and shoots to total uptake (Figure 7). Across treatments, roots accounted for the dominant share of total uptake for most metals, with As and Pb consistently exceeding 85% and Cd also surpassing 90% in hemp. For Cr, Ni, and Cu, root distributions were intermediate, generally ranging between ~70–85% in sorghum and ~50–65% in hemp, whereas Zn displayed the lowest root retention, reaching only up to ~60% in sorghum and ~35% in hemp.

At the treatment level, sorghum showed relatively stable root distributions for Ni, Zn, As, and Pb, with only modest differences among S, S/B, and S/F (≤10%). By comparison, Cr demonstrated higher root proportions in the inoculated variants, most notably in S/B. Elevated root shares were also identified for Cu in the inoculated variants, though the increases were smaller and more consistent than those for Cr. A distinct pattern was observed for Cd, with values rising from S to S/B but declining more clearly in S/F. Consistent with these modest shifts, none of the differences in root–shoot partitioning among S, S/B, and S/F were statistically significant for any metal (all *p* ≥ 0.067).

In hemp, root distributions for As, Cd, and Pb remained highly consistent across H, H/B, and H/F, with only minimal variation among treatments. Moderate shifts were observed for Cr and Ni, with slightly higher root fractions under inoculation but changes remained within a narrow overall range. More pronounced treatment-related effects were found for Cu, which demonstrated larger directional shifts across variants, and for Zn, where values increased markedly from H to H/B and decreased substantially in H/F relative to this peak. Despite the documented variation, none of the differences in root–shoot partitioning among H, H/B, and H/F were statistically significant for any metal (all *p* ≥ 0.25).

Across corresponding treatments, sorghum generally exhibited higher root contributions than hemp for most metals, particularly Cr, Ni, Cu, and Zn, whereas As and Pb showed similarly high root allocations in both species. For Cd, root proportions were consistently higher in hemp, with pronounced differences in S and S/F and more moderate divergence under bacterial inoculation.

#### 3.2.6. Translocation Factors

To complement the mass-based partitioning analysis, TF values are presented as a concentration-based descriptor of internal metal distribution and are summarised in Table 4. Across both plant species and all treatments, TFs remained consistently below unity (~0.01–0.33 in sorghum; ~0.01–0.53 in hemp), suggesting limited transfer of metals to aboveground tissues. The magnitude of TFs varied noticeably among elements, with Zn displaying the highest values in both species (~0.30–0.53), followed by intermediate levels for Cr, Ni, and Cu (~0.06–0.22). Lower TF values were detected for Cd (~0.01–0.15), whereas As and Pb systematically exhibited the lowest values across both species and treatments (~0.01–0.04).

At the treatment level, TFs in sorghum for Ni and Zn remained stable across S, S/B, and S/F, with only marginal variation between treatments (≤10%). By contrast, Cr and Cd showed reduced TFs in S/B, while their responses differed in S/F, with Cr remaining below S and Cd exceeding it. For Cu and Pb, TFs were lower in S/F than in both the S and S/B treatments. A distinct pattern was observed for As, with TFs increasing from S to S/B and declining again in S/F, without returning to S. Despite the described variation, none of the differences in TFs among S, S/B, and S/F were statistically significant for any metal (all *p* ≥ 0.061).

In hemp, Zn demonstrated largely stable TFs across H, H/B, and H/F, with only minor variation among treatments. For Cr and Ni, TFs were comparable between H and H/B and decreased in H/F, whereas Pb exhibited a progressive decline across H, H/B, and H/F. A non-monotonic pattern was observed for Cu, with TFs decreasing from H to H/B and increasing again in H/F, approaching H. A comparable response was recorded for Cd and As, as TFs increased from H to H/B and declined again in H/F, with Cd returning to H, while As remained above it. Despite the described variation, none of the differences in TFs among H, H/B, and H/F were statistically significant for any metal (all *p* ≥ 0.061).

Across corresponding treatments, hemp displayed higher TFs than sorghum for Zn, Ni, Cu, Cr, and As, while TFs for Cd and Pb were consistently lower in hemp, with Pb demonstrating smaller interspecies differences.

#### 3.2.7. Considerations for Interpreting Plant-Related Results

While variations in mean values were reported across plant-related parameters in the preceding sections, these findings should be viewed in light of the considerations outlined below. All plant-related results were derived from a limited number of replicates (n = 3 per treatment), which restricted statistical power. Consequently, nearly all treatment-level comparisons did not reach statistical significance, despite observable differences in mean values. In addition, biological variability among individual plants contributed to within-treatment dispersion, particularly under outdoor pot conditions. This variability is further propagated through ratio-based indices, which integrate uncertainty from both the numerator and denominator, whereas absolute uptake metrics incorporate biomass effects. To ensure consistent interpretation across all plant parameters, differences of ≤10% were regarded as marginal, and larger differences were reported qualitatively without causal inference. Accordingly, plant-related results are reported primarily in terms of relative magnitudes and variations in mean values, while broader system-level interpretation is provided in the Discussion.

## 4. Discussion

The present study assessed the potential influence of two PGPM inoculants on the behaviour of multiple heavy metals in a contaminated dredged sediment system cultivated with *Sorghum bicolor* L. and *Cannabis sativa* L. Rather than seeking to establish consistent enhancement of metal uptake, the study was designed to examine whether PGPM inoculation was associated with changes in plant metal accumulation and internal metal distribution under environmentally relevant outdoor pot conditions. Across the investigated parameters, the results were characterised by modest effects, substantial variability, and a general lack of statistical significance at the treatment level.

Such an overall result profile is not unexpected for outdoor pot experiments conducted on inherently heterogeneous dredged sediments, where plant development, microbial activity, and metal speciation are jointly governed by intrinsic substrate properties and biological interactions [45,46,47]. Within this context, parameters describing internal plant distribution of metals provide a particularly informative framework for interpreting metal dynamics across treatments and species.

Accordingly, the Discussion adopts a pattern-oriented approach, focusing on recurring trends across complementary metrics rather than isolated treatment effects or individual metals. Emphasis is placed on internal plant distribution of metals, including root–shoot concentration patterns and translocation behaviour, while PGPM-associated differences are addressed in terms of their magnitude and context dependence. Sediment-based metrics are employed mainly for environmental background, not for establishing direct causal links with plant uptake.

Across all treatments and both plant species, internal plant distribution of metals was consistently characterized by pronounced root retention and limited translocation to aboveground tissues. Root metal concentrations exceeded those in shoots for all investigated elements, and translocation factors remained below unity throughout, indicating a conservative internal handling strategy that was largely unaffected by PGPM inoculation. This root-dominant distribution pattern suggests that internal metal allocation was principally governed by intrinsic plant control rather than treatment-associated differences. Such behaviour is commonly recognized as indicative of a phytostabilization-type response, whereby metals are preferentially sequestered in belowground tissues [22,24,48,49]. In the context of the present study, this consistent internal distribution pattern provides a more robust grounding for interpretation than absolute uptake metrics, which were more susceptible to variability in plant biomass.

While concentration-based indices consistently indicated conservative internal metal handling, mass-based metrics revealed a more nuanced picture of metal allocation within the plants. Root–shoot partitioning and absolute metal uptake are inherently influenced by plant biomass and growth differences and therefore capture quantitative aspects of metal accumulation rather than regulatory allocation processes. Importantly, the coexistence of low TFs with less pronounced root-dominated mass-based root–shoot allocation can be attributed to differences between concentration-derived and mass-based metrics, as opposed to a shift toward greater translocation to aboveground tissues. Absolute metal uptake varied across treatments and between the two plant species, largely reflecting differences in biomass production instead of uniform shifts in internal metal allocation. Taken together, these findings highlight the value of jointly considering concentration-based indices and mass-based metrics when assessing plant metal behaviour in contaminated sediment systems [10,12,14].

Against this background of strong intrinsic control over internal metal allocation, the responses to PGPM inoculation in terms of absolute plant metal uptake were generally modest and variable, without evidence of consistent or directional enhancement across species and treatments. Within this quantitative framework, PGPM-associated differences in absolute metal uptake were restricted to variation in magnitude, without indicating a systematic modification of accumulation behaviour. Notably, the direction and extent of these uptake variations differed between the two plant species, suggesting that species identity conditioned the expression of PGPM-associated differences.

Beyond treatment-related variability, species identity emerged as a dominant factor structuring absolute metal uptake, with sorghum consistently exhibiting higher uptake magnitudes than hemp across corresponding treatments. This interspecific contrast was primarily captured by differences in uptake magnitude, reflecting differences in plant biomass and growth characteristics rather than divergent metal handling strategies. Accordingly, the higher absolute uptake observed in sorghum does not imply a shift toward phytoextraction-type behaviour, but instead reflects a higher capacity for metal retention consistent with its larger biomass [11,12,13,50].

Despite the noted differences in plant metal uptake across species and treatments, the absolute amounts accumulated in plant tissues accounted for only a minor fraction of the total metal pools present in the dredged sediment, with even the highest values falling below approximately 0.2%. In this context, any measurable changes observed in sediment metal concentrations over the course of the experiment cannot be directly ascribed to plant accumulation alone, but are likely associated with the combined influence of sediment heterogeneity, physical redistribution, and geochemical re-equilibration processes operating at the system scale [45,46,51,52].

Beyond total sediment concentrations, BCR fractionation offered additional qualitative insight into the environmental context of metal behaviour within the dredged sediment system. In general, baseline fractionation profiles indicated that metals were dominantly associated with low-mobility fractions, consistent with a sediment matrix characterised by limited immediate metal availability despite elevated pseudo-total concentrations. Following plant growth, shifts among fractions were primarily reflected as moderate internal redistributions between residual, reducible, and oxidisable pools, with the exchangeable fraction remaining largely unchanged. Importantly, the magnitude of these redistributions was overall limited and frequently within the range of analytical uncertainty, indicating that the chemical stability of the sediment was not fundamentally altered over the course of the experiment. Taken together, these patterns characterize a chemically conservative sediment environment, in which plant- and treatment-related responses were expressed under conditions of intrinsically constrained metal mobility [10,21,53,54].

Within this geochemical setting, BAFs serve as dimensionless descriptors of relative sediment-to-plant metal transfer, whose interpretive value is fundamentally system dependent. Accordingly, higher BAFs in roots than in shoots indicated a root-dominated accumulation pattern that is fully aligned with the conservative internal metal handling and limited translocation documented across species and treatments. However, when assessed alongside mass-based uptake metrics, it becomes clear that moderate BAFs do not necessarily correspond to substantial metal removal, particularly in sediment systems characterized by elevated total metal pools. This divergence between relative accumulation and system-scale removal is particularly apparent in the present study, where plant- associated metal pools remained negligible with respect to the total sediment metal burden. These observations underscore the necessity of interpreting BAFs jointly with mass-based metrics when evaluating plant metal behaviour in severely contaminated sediment systems [9,11,14,55].

To relate the present BAF and TF values to previously reported data, selected values for plants grown in contaminated sediments and related contaminated substrates are summarized in Table 5. Because these indices are strongly affected by substrate properties, contamination level, plant species, cultivation duration, and treatment strategy, the comparison should be interpreted descriptively rather than as a direct performance ranking. Nevertheless, several recurring patterns can be identified. Most notably, BAF values are frequently higher in roots than in shoots, while TF values often remain below unity, consistent with preferential metal retention in belowground tissues rather than extensive phytoextraction. These cross-study patterns support the phytostabilization-oriented interpretation of the present results [10,21,22,45,49,51,54].

In a broader context, PGPMs, encompassing both bacterial and fungal inoculants, have been recognized to influence plant–metal interactions through multiple rhizosphere-mediated pathways. Such pathways have been described to include alterations in rhizosphere chemistry (e.g., pH and organic ligand exudation), microbial metal complexation, and indirect effects on root development. However, the extent to which such pathways are expressed is highly context dependent, governed by sediment geochemistry, plant species traits, and system-level constraints, and therefore may not manifest uniformly across experimental settings [52,56,57,58]. Within this framework, the present findings indicate that plant species identity, biomass production, root-dominated metal retention, and sediment constraints were more prominent determinants of metal uptake and allocation than the context-dependent and secondary PGPM-associated responses, supporting a phytostabilization-oriented interpretation for both species under the investigated dredged sediment conditions.

### Limitations and Future Research Perspectives

In addition to the considerations outlined in Section 3.2.7, the broader interpretation of the present findings must account for several system-level and methodological constraints. Although the outdoor pot design using real dredged sediment enhanced environmental relevance, it inevitably reduced the degree of experimental control over sediment heterogeneity and fine-scale geochemical variability. Nevertheless, the pot-based setup remains a simplified experimental system that does not fully reproduce field-scale hydrology, rooting conditions, or long-term environmental dynamics. Furthermore, irrigation was managed to avoid excessive wetting and visible leaching; however, substrate moisture was not continuously instrument-monitored, and leachate was not collected or chemically analysed. As a result, potential water-driven changes in metal mobility, redistribution, or leaching losses could not be directly quantified. The relatively short 10-week cultivation period also limits inference on longer-term metal stabilization, exhaustion of sediment metal pools, temporal changes in metal binding, and persistence of sediment–plant–PGPM interactions. Finally, while shifts among BCR fractions and plant-related parameters were documented, the present design does not permit causal attribution of these changes to specific biological or physicochemical processes, constraining extrapolation beyond comparable dredged sediment systems [13,15,21,23,59].

Future research should build on the present system-level assessment by examining the persistence and scalability of phytostabilization-oriented responses under field-relevant conditions. Key aspects to evaluate include whether root-dominated metal retention remains stable across extended or repeated cultivation cycles, temporal changes in sediment metal binding, the influence of water dynamics on metal mobility, and direct characterization of PGPM establishment and rhizosphere-level processes.

From an applied perspective, future studies should assess whether the modest and context-dependent PGPM-associated responses observed here can be enhanced or made more consistent through integrated amendment-based management strategies. These may include biochar-based approaches derived from harvested biomass, as well as other organic amendment strategies, alone or in combination with PGPM inoculation [24,59]. Such work would help determine how the phytostabilization-oriented responses observed in this study can be translated into practical recultivation and management strategies for contaminated dredged sediments.

## 5. Conclusions

This study evaluated whether bacterial and fungal PGPM inoculation was associated with changes in plant metal uptake and internal allocation in a contaminated dredged sediment system cultivated with *Sorghum bicolor* L. and *Cannabis sativa* L. Across the evaluated parameters, PGPM inoculation resulted in overall modest and variable responses, without evidence of consistent or directional enhancement of metal uptake across species or treatments. Instead, intrinsic plant traits shaped metal behaviour, with both species showing conservative internal handling characterized by dominant root retention and limited translocation, compatible with a phytostabilization-type response. At the system scale, absolute metal uptake by plants represented only a minor fraction of the total sediment metal pools, suggesting that phytoremediation outcomes were constrained by the magnitude and geochemical stability of the dredged sediment matrix. Taken together, these results imply that in heterogeneous dredged sediment systems, expectations of PGPM-assisted phytoremediation should be calibrated toward context-dependent modulation and stabilization rather than systematic facilitation of metal extraction.

## Figures and Tables

**Table 1:** Physicochemical properties of the dredged sediment used in the pot experiment.

Parameter	Unit	Value
**pH**	/	7.92 ± 0.18
**Electrical conductivity**	µS/cm	211.33 ± 35.87
**Organic matter**	%	7.91 ± 1.02
**Total nitrogen**	%	0.22 ± 0.01
**Total phosphorus**	%	0.060 ± 0.01
**Total potassium**	%	0.33 ± 0.04
**Cation exchange capacity**	cmol_(c)_/kg	64.39 ± 26.10
**Texture**	% Sand (50–2000 µm)	79.91 ± 1.37
	% Silt (2–50 µm)	6.95 ± 0.27
	% Clay (<2 µm)	13.14 ± 1.13

**Table 2:** Identified within-treatment trends in total metal mass (mg per pot) based on pre- and post-harvest concentrations.

Treatment Code	Metal Trend
**S**	Zn > Cr > Cu > Ni > Pb > As > Cd
**S/B**	Zn > Cr > Cu > Ni > As > Cd > Pb
**S/F**	Cu > Ni > As > Cr > Cd > Zn > Pb
**H**	Cu > Zn > Cr > Ni > As > Cd > Pb
**H/B**	Zn > Cr > Cu > Ni > As > Cd > Pb
**H/F**	Ni > Zn > Cu > Cr > As > Pb > Cd

**Table 3:** Bioaccumulation factors for roots and shoots across all investigated treatments. Values are presented as mean ± SD (n = 3).

Metal	Treatment Code					
	S	S/B	S/F	H	H/B	H/F
**Cr**	0.25 ± 0.08	0.12 ± 0.01	0.16 ± 0.01	0.13 ± 0.02	0.10 ± 0.01	0.12 ± 0.01
	2.40 ± 0.81	2.01 ± 0.37	2.03 ± 0.27	0.66 ± 0.21	0.54 ± 0.08	0.99 ± 0.06
**Ni**	0.28 ± 0.06	0.23 ± 0.03	0.22 ± 0.01	0.18 ± 0.03	0.15 ± 0.02	0.21 ± 0.03
	2.11 ± 0.85	1.44 ± 0.28	1.37 ± 0.09	0.86 ± 0.23	0.74 ± 0.11	1.23 ± 0.15
**Cu**	0.08 ± 0.03	0.09 ± 0.01	0.08 ± 0.02	0.12 ± 0.04	0.09 ± 0.00	0.14 ± 0.03
	0.67 ± 0.08	0.74 ± 0.14	0.91 ± 0.18	0.60 ± 0.16	0.59 ± 0.16	0.68 ± 0.09
**Zn**	0.30 ± 0.16	0.30 ± 0.05	0.30 ± 0.05	0.29 ± 0.04	0.36 ± 0.01	0.45 ± 0.13
	0.96 ± 0.26	0.98 ± 0.26	0.96 ± 0.17	0.63 ± 0.29	0.78 ± 0.15	0.86 ± 0.09
**As**	0.02 ± 0.02	0.03 ± 0.02	0.04 ± 0.01	0.04 ± 0.02	0.07 ± 0.01	0.04 ± 0.03
	2.29 ± 0.39	1.99 ± 1.76	3.47 ± 1.12	2.29 ± 1.04	3.31 ± 1.46	2.69 ± 1.37
**Cd**	0.22 ± 0.15	0.15 ± 0.06	0.24 ± 0.05	0.02 ± 0.01	0.03 ± 0.01	0.03 ± 0.01
	1.78 ± 0.42	1.76 ± 0.65	1.58 ± 0.12	1.33 ± 0.21	1.56 ± 0.65	1.92 ± 0.28
**Pb**	0.27 ± 0.02	0.27 ± 0.16	0.21 ± 0.06	0.31 ± 0.10	0.28 ± 0.10	0.68 ± 0.25
	0.01 ± 0.01	0.01 ± 0.00	0.01 ± 0.00	0.01 ± 0.01	0.01 ± 0.00	0.01 ± 0.00

Shoot and root BAFs are presented as stacked rows for each metal. Values shown as 0.00 indicate SDs smaller than the applied rounding precision.

**Table 4:** Translocation factors across all investigated treatments. Values are reported as mean ± SD (n = 3).

Metal	Treatment Code					
	S	S/B	S/F	H	H/B	H/F
**Cr**	0.12 ± 0.08	0.06 ± 0.01	0.08 ± 0.01	0.20 ± 0.03	0.19 ± 0.02	0.12 ± 0.02
**Ni**	0.15 ± 0.07	0.17 ± 0.04	0.16 ± 0.01	0.22 ± 0.04	0.21 ± 0.04	0.17 ± 0.04
**Cu**	0.12 ± 0.06	0.13 ± 0.04	0.09 ± 0.02	0.21 ± 0.08	0.16 ± 0.05	0.21 ± 0.08
**Zn**	0.29 ± 0.09	0.33 ± 0.10	0.32 ± 0.07	0.52 ± 0.16	0.48 ± 0.11	0.53 ± 0.20
**As**	0.01 ± 0.01	0.03 ± 0.03	0.01 ± 0.00	0.02 ± 0.00	0.03 ± 0.02	0.02 ± 0.02
**Cd**	0.12 ± 0.05	0.09 ± 0.03	0.15 ± 0.05	0.01 ± 0.01	0.02 ± 0.01	0.01 ± 0.00
**Pb**	0.04 ± 0.02	0.04 ± 0.02	0.03 ± 0.01	0.04 ± 0.01	0.03 ± 0.01	0.02 ± 0.01

Values shown as 0.00 indicate SDs smaller than the applied rounding precision.

**Table 5:** Comparison of selected BAF and TF values reported for plants grown in contaminated sediments and related contaminated substrates.

Plant Species	Substrate	Treatment	Metal	BAF (Shoot)	BAF (Root)	TF Range	Ref.
*Sorghum bicolor*	Dredged sediment (Bega Canal)	Organic acids (GA, TA)	**Cr**	0.026–0.360	0.402–1.652	0.024–0.583	[21]
			**Ni**	0.065–0.302	0.228–0.511	0.040–0.253	
			**Cu**	0.077–0.176	14.97–20.79	0.172–0.373	
			**Cd**	0.879–2.804	1.298–1.825	0.524–1.616	
			**Pb**	0.007–0.025	0.157–0.419	0.019–0.107	
*Cannabis sativa*			**Cr**	0.006–0.048	0.031–0.140	0.199–0.926	
			**Ni**	0.050–0.137	0.018–0.336	0.349–0.771	
			**Cu**	0.048–0.150	0.175–4.321	0.602–1.065	
			**Cd**	0.029–0.117	0.159–0.519	0.120–0.338	
			**Pb**	0.004–0.022	0.009–0.096	0.237–3.107	
*Typha angustifolia*	Dredged sediment	Aeration, waterlogged	**Cr**	0.020–0.032	0.088–0.090	0.226–0.357	[10]
			**Pb**	0.026–0.054	0.063–0.148	0.365–0.539	
			**Cu**	0.181–0.237	0.313–0.576	0.397–0.578	
			**Cd**	n.d.	0.314–0.327	n.d.	
*Brassica napus*	Dredged sediment (Bega Canal)	Organic acids, N–fertilizers	**Cr**	0.011–0.054	n.a.	0.319–1.395	[54]
			**Cu**	0.063–0.212	n.a.	0.359–2.007	
			**Cd**	0.271–0.460	n.a.	1.023–2.916	
			**Pb**	0.008–0.037	n.a.	0.230–1.192	
*Cannabis sativa*	Contaminated soil	PGPR, EDTA	**Cr**	0.003–0.004	0.07–0.08	0.03–0.06	[22]
			**Ni**	0.03–0.29	0.21–0.29	0.13–1.07	
			**Cu**	0.08–0.49	0.30–0.65	0.27–0.77	
*Sorghum bicolor*	Arid soil	MAC levels	**Co**	0.976–1.374	1.034–1.811	0.539–1.012	[49]
			**Cd**	0.154–0.302	0.166–0.533	0.566–1.020	
			**Pb**	0.348–0.597	0.569–0.937	0.372–0.965	
*Spartium junceum*	Dredged marine sediment	Compost, plant combinations	**Cu**	~0.25	~0.33	~0.72	[45]
			**Cd**	~0.70	~0.79	~0.87	
			**Ni**	~0.02	~0.15	~0.23	
			**Zn**	~0.21	~0.20	~1.01	
			**Pb**	~0.02	~0.05	~0.32	
*Tamarix gallica*			**Cu**	~0.30	~0.52	~0.55	
			**Cd**	~0.62	~0.88	~0.70	
			**Ni**	~0.05	~0.08	~0.06	
			**Zn**	~0.15	~0.14	~1.02	
			**Pb**	~0.02	~0.11	~0.10	
*Noccaea caerulescens*	Contaminated soil	None	**Cd**	102.5	15.5	6.6	[51]
			**Zn**	39.3	16.1	2.5	
			**Pb**	0.1	0.2	0.4	
			**Cu**	0.3	0.7	0.4	

**Note:** Present-study BAF and TF values are given in Table 3 and Table 4, respectively; Ref.—reference; n.d.—not detected; n.a.—not available; Ref. [54] reports shoot BAF values only.

## Data Availability

The original contributions presented in this study are included in the article/Supplementary Materials. Further inquiries can be directed to the corresponding author.

## Supplementary Materials

The following supporting information can be downloaded at: https://www.mdpi.com/article/10.3390/jox16030102/s1, Table S1: Cultivar, source, and seed-lot quality parameters of *Sorghum bicolor* L. and *Cannabis sativa* L. used in the pot experiment; Table S2: Daily meteorological parameters during the 10-week cultivation period [26]. Table S3: Element-specific AAS conditions and validated method-performance parameters for heavy metal determination.

## References

[1] Alfee S.L., Bloor M.C. (2025). A Global Review of River Sediment Contamination and Remobilization through Climate Change-Induced Flooding. Sustain. Environ..

[2] Dorleon G., Rigaud S., Techer I. (2025). Management of Dredged Marine Sediments in Southern France: Main Keys to Large-scale Beneficial Re-use. Environ. Sci. Pollut. Res..

[3] Montigny C., Chouba C., Domeau A., Delpoux S., Marie M., Causse L., Freydier R., Pringault O. (2025). Assessing Pollution in Sediment and Water before, during and after Sediment Dredging in a Mediterranean Harbor. J. Environ. Manag..

[4] Welsch M., Bentsen S., Henning M. (2025). Focused Review of Recent Advances of Sediment Treatment Technologies. Integr. Environ. Assess. Manag..

[5] Beljin J., Arsenov D., Slijepčcević N., Maletić S., Đukanović N., Chalot M., Županski M., Tomašević Pilipović D. (2023). Recycling of Polluted Dredged Sediment—Building New Materials for Plant Growing. Waste Manag..

[6] Afolayan A., Černý R., Fořt J. (2025). Review of Treatment Techniques for Dredged Sediments in the Context of Valorization as Secondary Raw Materials. Buildings.

[7] Sun C., Gong W., Pan G., Mortimer R.J.G., Yao E., Wen S., Chen M., Zhong J. (2025). Comprehensive Effects of Sediment Dredging on Environmental Risk and Bioavailability of Heavy Metals from the Sediment of Lake Taihu, China. J. Hazard. Mater..

[8] Wu G., Reichart G.-J., Kraal P. (2025). Reactivity and Potential Mobility of Metals in Human-Impacted Harbor Sediments (Port of Rotterdam, the Netherlands). J. Soils Sediments.

[9] Diarra I., Kotra K.K., Prasad S. (2021). Application of Phytoremediation for Heavy Metal Contaminated Sites in the South Pacific: Strategies, Current Challenges and Future Prospects. Appl. Spectrosc. Rev..

[10] Wu J., Yang L., Zhong F., Cheng S. (2014). A Field Study on Phytoremediation of Dredged Sediment Contaminated by Heavy Metals and Nutrients: The Impacts of Sediment Aeration. Environ. Sci. Pollut. Res..

[11] Wood J.L., Tang C., Franks A.E. (2016). Microbial Associated Plant Growth and Heavy Metal Accumulation to Improve Phytoextraction of Contaminated Soils. Soil Biol. Biochem..

[12] He X., Xu M., Wei Q., Tang M., Guan L., Lou L., Xu X., Hu Z., Chen Y., Shen Z. (2020). Promotion of Growth and Phytoextraction of Cadmium and Lead in *Solanum nigrum* L. Mediated by Plant-Growth-Promoting Rhizobacteria. Ecotoxicol. Environ. Saf..

[13] Bai X., Bol R., Chen H., Cui Q., Qiu T., Zhao S., Fang L. (2024). A Meta-Analysis on Crop Growth and Heavy Metals Accumulation with PGPB Inoculation in Contaminated Soils. J. Hazard. Mater..

[14] Eben P., Mohri M., Pauleit S., Duthweiler S., Helmreich B. (2024). Phytoextraction Potential of Herbaceous Plant Species and the Influence of Environmental Factors—A Meta-Analytical Approach. Ecol. Eng..

[15] Abdollahi S., Golchin A., Shahryari F., Alamdari P. (2020). PGPR Inoculation of a Contaminated Soil Affects Plant Growth and Phytoavailability of Cd and Pb. Arch. Agron. Soil Sci..

[16] Muratova A., Golubev S., Romanova V., Sungurtseva I., Nurzhanova A. (2023). Effect of Heavy-Metal-Resistant PGPR Inoculants on Growth, Rhizosphere Microbiome and Remediation Potential of *Miscanthus* × *Giganteus* in Zinc-Contaminated Soil. Microorganisms.

[17] Hnini M., Rabeh K., Oubohssaine M. (2024). Interactions between Beneficial Soil Microorganisms (PGPR and AMF) and Host Plants for Environmental Restoration: A Systematic Review. Plant Stress.

[18] Iqbal M.Z., Singh K., Chandra R. (2024). Recent Advances of Plant Growth Promoting Rhizobacteria (PGPR) for Eco-Restoration of Polluted Soil. Clean. Eng. Technol..

[19] Qin H., Wang Z., Sha W., Song S., Qin F., Zhang W. (2024). Role of Plant-Growth-Promoting Rhizobacteria in Plant Machinery for Soil Heavy Metal Detoxification. Microorganisms.

[20] Dubovina M., Krčmar D., Grba N., Watson M.A., Rađenović D., Tomašević-Pilipović D., Dalmacija B. (2018). Distribution and Ecological Risk Assessment of Organic and Inorganic Pollutants in the Sediments of the Transnational Begej Canal (Serbia-Romania). Environ. Pollut..

[21] Ðukanović N., Beljin J., Zeremski T., Stojanov N., Milić S., Kragulj Isakovski M., Maletić S. (2025). Phytoremediation Efficiency of Hemp and *Sorghum* Grown in Contaminated Sediment: The Role of Organic Acids. Agronomy.

[22] Ferrarini A., Fracasso A., Spini G., Fornasier F., Taskin E., Fontanella M.C., Beone G.M., Amaducci S., Puglisi E. (2021). Bioaugmented Phytoremediation of Metal-Contaminated Soils and Sediments by Hemp and Giant Reed. Front. Microbiol..

[23] Moreira H., Pereira S.I.A., Mench M., Garbisu C., Kidd P., Castro P.M.L. (2021). Phytomanagement of Metal(Loid)-Contaminated Soils: Options, Efficiency and Value. Front. Environ. Sci..

[24] Anbuganesan V., Vishnupradeep R., Mehnaz N., Kumar A., Freitas H., Rajkumar M. (2024). Synergistic Effect of Biochar and Plant Growth Promoting Bacteria Improve the Growth and Phytostabilization Potential of *Sorghum bicolor* in Cd and Zn Contaminated Soils. Rhizosphere.

[25] Bajić I., Pejić B., Sikora V., Kostić M., Ivanovska A., Pejić B., Vojnov B. (2022). The Effects of Irrigation, Topping, and Interrow Spacing on the Yield and Quality of Hemp (*Cannabis sativa* L.) Fibers in Temperate Climatic Conditions. Agriculture.

[26] Meteostat Historical Weather and Climate Data. https://meteostat.net/.

[27] Huang W., Zhang C., Zhu B., Liu X., Xiao H., Liu S., Shao H. (2025). Systematic Evaluation of Plant Metals/Metalloids Accumulation Efficiency: A Global Synthesis of Bioaccumulation and Translocation Factors. Front. Plant Sci..

[28] Roebuck C.J., Klink M.J. (2025). Phytoremediation Potential of Hemp in Metal-Contaminated Soils: Soil Analysis, Metal Uptake, and Growth Dynamics. Processes.

[29] (2005). Soil Quality—Determination of PH.

[30] (1994). Soil Quality—Determination of the Specific Electrical Conductivity.

[31] (2000). Characterization of Sludges—Determination of the Loss on Ignition of Dry Mass.

[32] (1995). Soil Quality—Determination of Total Nitrogen—Modified Kjeldahl Method.

[33] American Public Health Association, American Water Works Association, Water Environment Federation (1997). Standard Methods for the Examination of Water and Wastewater.

[34] van Reeuwijk L.P. (2002). Procedures for Soil Analysis.

[35] U.S. EPA (1998). Method 7010: Graphite Furnace Atomic Absorption Spectrophotometry. Test Methods for Evaluating Solid Waste, Physical/Chemical Methods (SW-846).

[36] (2009). Soil Quality—Determination of Particle Size Distribution in Mineral Soil Material—Method by Sieving and Sedimentation.

[37] U.S. EPA (2007). Method 3051A: Microwave Assisted Acid Digestion of Sediments, Sludges, Soils, and Oils. Test Methods for Evaluating Solid Waste, Physical/Chemical Methods (SW-846).

[38] U.S. EPA (2007). Method 7000B: Flame Atomic Absorption Spectrophotometry. Test Methods for Evaluating Solid Waste, Physical/Chemical Methods (SW-846).

[39] Arain M.B., Kazi T.G., Jamali M.K., Jalbani N., Afridi H.I., Baig J.A. (2008). Speciation of Heavy Metals in Sediment by Conventional, Ultrasound and Microwave Assisted Single Extraction Methods: A Comparison with Modified Sequential Extraction Procedure. J. Hazard. Mater..

[40] Abed S.N., Almuktar S.A., Scholz M. (2019). Phytoremediation Performance of Floating Treatment Wetlands with Pelletized Mine Water Sludge for Synthetic Greywater Treatment. J. Environ. Health Sci. Eng..

[41] Dietrich C.C., Tandy S., Murawska-Wlodarczyk K., Banaś A., Korzeniak U., Seget B., Babst-Kostecka A. (2021). Phytoextraction Efficiency of Arabidopsis Halleri Is Driven by the Plant and Not by Soil Metal Concentration. Chemosphere.

[42] Paniagua-López M., García-Robles H., Aguilar-Garrido A., Romero-Freire A., Lorite J., Sierra-Aragón M. (2024). Vegetation Establishment in Soils Polluted by Heavy Metal(Loid)s after Assisted Natural Remediation. Plant Soil.

[43] Government of the Netherlands Regeling Bodemkwaliteit (2022). Bijlage B, Tabel 1: Normwaarden Voor Toepassen van Grond of Baggerspecie Op of in de Bodem. https://wetten.overheid.nl/BWBR0047808/2026-01-31#BijlageB.

[44] Vieira D.C.S., Yunta F., Baragaño D., Evrard O., Reiff T., Silva V., de la Torre A., Zhang C., Panagos P., Jones A. (2024). Soil Pollution in the European Union—An Outlook. Environ. Sci. Policy.

[45] Doni S., Macci C., Peruzzi E., Iannelli R., Masciandaro G. (2015). Heavy Metal Distribution in a Sediment Phytoremediation System at Pilot Scale. Ecol. Eng..

[46] Ferrans L., Schmieder F., Mugwira R., Marques M., Hogland W. (2022). Dredged Sediments as a Plant-Growing Substrate: Estimation of Health Risk Index. Sci. Total Environ..

[47] Sandhi A., Gao L., Rosenlund J., Landberg T. (2023). Growing *Salix* spp. on Heavy Metal Contaminated Sediment (Oskarshamn, Sweden) as a Joint Phytoremediation and Circular Economy Approach. Environ. Adv..

[48] Soudek P., Petrová Š., Vaňková R., Song J., Vaněk T. (2014). Accumulation of Heavy Metals Using *Sorghum* sp.. Chemosphere.

[49] Sagimbayeva A.M., Tomlekova N.B., Saparov G.A., Abduraimov Y.O., Kerimbayev A.A., Nurabayev S.S., Assanzhanova N.N., Akmyrzayev N.Z., Iskakova K.M., Omarova A.S. (2025). Phytoremediation of Heavy Metal-Contaminated Soil Using Drought-Adapted Sweet Sorghum (*Sorghum bicolor* L.) in Arid Regions of Kazakhstan. Plants.

[50] Tan Q., Guo Q., Wei R., Zhu G., Du C., Hu H. (2023). Influence of Arbuscular Mycorrhizal Fungi on Bioaccumulation and Bioavailability of As and Cd: A Meta-Analysis. Environ. Pollut..

[51] He W., Long A., Zhang C., Cao M., Luo J. (2021). Mass Balance of Metals during the Phytoremediation Process Using Noccaea Caerulescens: A Pot Study. Environ. Sci. Pollut. Res..

[52] Deng S., Zhang X., Zhu Y., Zhuo R. (2024). Recent Advances in Phyto-Combined Remediation of Heavy Metal Pollution in Soil. Biotechnol. Adv..

[53] Ferrans L., Jani Y., Burlakovs J., Klavins M., Hogland W. (2021). Chemical Speciation of Metals from Marine Sediments: Assessment of Potential Pollution Risk While Dredging, a Case Study in Southern Sweden. Chemosphere.

[54] Stojanov N., Maletić S., Beljin J., Đukanović N., Kiprovski B., Zeremski T. (2024). Enhancing Phytoextraction Potential of Brassica Napus for Contaminated Dredged Sediment Using Nitrogen Fertilizers and Organic Acids. Plants.

[55] Kumar A., Tripti, Raj D., Maiti S.K., Maleva M., Borisova G. (2022). Soil Pollution and Plant Efficiency Indices for Phytoremediation of Heavy Metal(Loid)s: Two-Decade Study (2002–2021). Metals.

[56] Alves A.R.A., Yin Q., Oliveira R.S., Silva E.F., Novo L.A.B. (2022). Plant Growth-Promoting Bacteria in Phytoremediation of Metal-Polluted Soils: Current Knowledge and Future Directions. Sci. Total Environ..

[57] Wang Y., Narayanan M., Shi X., Chen X., Li Z., Natarajan D., Ma Y. (2022). Plant Growth-Promoting Bacteria in Metal- Contaminated Soil: Current Perspectives on Remediation Mechanisms. Front. Microbiol..

[58] Montreemuk J., Stewart T.N., Prapagdee B. (2024). Bacterial-Assisted Phytoremediation of Heavy Metals: Concepts, Current Knowledge, and Future Directions. Environ. Technol. Innov..

[59] Xiong Y., Lin R., Wang Y., Liu K., Guo J., Wu M., Chen Q., Oleszczuk P., Pan B. (2026). Selective Application of Biochars to Realize Biochar–Microbe Synergistic Immobilization of Soil Cadmium. Environ. Biogeochem. Process..

