# Supplementary material for: Sorghum and Hemp Responses to Plant Growth-Promoting Microorganism Inoculation in Metal-Contaminated Dredged Sediment: A System-Level Assessment Under Environmentally Relevant Outdoor Pot Conditions
Source: J Xenobiot. 2026 Jun 2;16(3):102. doi: 10.3390/jox16030102 (PMC13302178; doi:10.3390/jox16030102)
Supplement: Supplementary file 1 [file jox-16-00102-s001.zip › jox-4293427-supplementary.pdf]

Journal of *Xenobiotics*

MDPI

Sorghum and Hemp Responses to Plant Growth-Promoting Microorganism Inoculation in Metal-Contaminated Dredged Sediment: A System-Level Assessment Under Environmentally Relevant Outdoor Pot Conditions

Marko Šolić, Nina Đukanović, Tamara Apostolović, Jelena Beljin, Irina Jevrosimov, Dragana Tamindžija, Ivana Bajić, Stanko Milić, Tijana Zeremski, Marijana Kragulj Isakovski and Snežana Maletić

Table S1. Cultivar, source, and seed-lot quality parameters of *Sorghum bicolor* L. and *Cannabis sativa* L. used in the pot experiment.

**Parameter**	***Sorghum bicolor* L.**	***Cannabis sativa* L.**
**Cultivar/variety**	NS Džin	HELENA
**Seed category**	Pre-basic seed	C1
**Production year**	2024	2020
**Producer**	IFVCNS	IFVCNS
**Processor**	IFVCNS	IFVCNS
**Purity (%)**	99.8	99.9
**Germination (%)**	88	70
**Germination energy (%)**	87	63
**Moisture content (%)**	14.6	10.3
**1000-seed weight (g)**	11.19	10.71
**Seed treatment**	Not specified	Not treated
**Health status**	*Alternaria* sp. 4%	Correct/satisfactory

Note: IFVCNS–Institute of Field and Vegetable Crops, Novi Sad, Serbia

 https://doi.org/10.3390/jox16030102

Table S2. Daily meteorological parameters during the 10-week cultivation period [1].

Date	T_{avg} (°C)	T_{min} (°C)	T_{max} (°C)	Prcp (mm)	W_{spd} (km/h)	W_{pgt} (km/h)	Pres hPa	t_{sun} (min)
**2024-04-25**	9.5	3.4	15.6	0.8	5.7	16.7	1009.7	353
**2024-04-26**	12.1	3.2	19.2	0	7.8	22.2	1013.3	505
**2024-04-27**	15.9	7.6	22.5	0	6.9	18.5	1017.2	575
**2024-04-28**	19.1	9.1	27	0	14.7	33.3	1020.4	660
**2024-04-29**	19.2	10	27.1	0	17.8	47	1022.8	662
**2024-04-30**	18.5	10.7	23.9	0	25.4	61	1023.3	614
**2024-05-01**	18.4	13.4	24.1	0	30.3	65	1016.1	504
**2024-05-02**	19.2	14.8	25.8	0	18.4	43	1010.3	276
**2024-05-03**	14.2	11.7	16.6	7.1	9.6	43	1008.9	0
**2024-05-04**	14.9	11.4	21.8	11.9	10.4	22.2	1012.7	391
**2024-05-05**	19.8	11.5	26.6	0	5.4	16.7	1014.3	639
**2024-05-06**	20.6	11.3	26.4	0.5	8.5	25.9	1012.4	571
**2024-05-07**	19.8	14.1	27.8	0	9.3	27.8	1012.8	379
**2024-05-08**	18.2	13.6	23.9	0.3	9	27.8	1016	231
**2024-05-09**	17.1	11.5	23	0	10.1	27.8	1019.3	382
**2024-05-10**	16.8	11.6	22	0	12.8	29.6	1019.9	455
**2024-05-11**	17.6	9.3	25.7	0	8.1	24.1	1018.8	533
**2024-05-12**	16.9	13	23.8	0	8	40	1018.2	278
**2024-05-13**	16.2	6.8	23.4	8.4	6.7	20.4	1018	679
**2024-05-14**	15.9	7.9	22.2	0	8.8	18.5	1014.3	418
**2024-05-15**	15.6	9.4	21.5	0	18.9	47	1015.7	572
**2024-05-16**	16.7	11.7	22.6	0	29.3	61	1014.1	378
**2024-05-17**	18.7	13.5	24.9	1	28	68	1011.1	350
**2024-05-18**	20.4	10.7	27.3	1.8	7.3	29.6	1014.2	578
**2024-05-19**	22.5	14.4	29.9	0	10.8	22.2	1011.5	439
**2024-05-20**	22.1	17.1	27.4	0	13	27.8	1012	389
**2024-05-21**	20.8	17.2	28	0	21.3	54	1009.7	359
**2024-05-22**	19.6	14.5	26.3	18.8	8.3	31.5	1010.8	408
**2024-05-23**	16.6	14.8	22.8	3.6	8.6	40	1015.1	188
**2024-05-24**	18.4	15.8	23.5	18	7.4	20.4	1018.6	295
**2024-05-25**	20	12.1	26.9	4.1	13.5	40	1017.3	603
**2024-05-26**	21.1	13.8	26.7	0	13.6	33.3	1017.6	507
**2024-05-27**	20	12.9	27.9	0	7.1	47	1018.7	648
**2024-05-28**	20.5	13	26.5	0	8.6	20.4	1014.7	636
**2024-05-29**	20.1	15.1	26.4	0.8	13.8	40	1012.8	505
**2024-05-30**	21.1	13.8	28.2	1.3	7.6	18.5	1007.9	525

https://doi.org/10.3390/jox16030102

Date	T_{avg} (°C)	T_{min} (°C)	T_{max} (°C)	Prcp (mm)	W_{spd} (km/h)	W_{pgt} (km/h)	Pres hPa	t_{sun} (min)
**2024-05-31**	21.6	14.8	25.2	0.5	13.9	40	1007.2	341
**2024-06-01**	23.6	15.7	30.6	0	10.2	29.6	1010.8	578
**2024-06-02**	23.1	14.3	30	0	5.9	18.5	1015.3	668
**2024-06-03**	19.6	16.2	25	5.8	8.5	61	1013.4	262
**2024-06-04**	18.2	12.7	24.2	15.2	12	43	1013.2	394
**2024-06-05**	20.8	13.9	26.6	0	5	16.7	1015.1	445
**2024-06-06**	23.2	14.3	30	0.8	5.2	13	1017	712
**2024-06-07**	26	16.4	33.2	0	6.2	16.7	1015.8	656
**2024-06-08**	25.3	18.3	32.4	0	6.3	16.7	1013.5	628
**2024-06-09**	26.5	21.5	31.6	0	10.8	29.6	1008.8	568
**2024-06-10**	26.1	20.9	34	0	10.3	24.1	1005.2	382
**2024-06-11**	21.1	19	25	14.2	8.5	24.1	1009.9	302
**2024-06-12**	19.3	17	23	1.8	10.2	25.9	1013.4	135
**2024-06-13**	17.8	14.4	22.1	1.5	8.4	47	1015.8	146
**2024-06-14**	18.3	12.3	24.6	9.9	10.5	25.9	1016.2	600
**2024-06-15**	20.4	12.3	28.2	0	5.7	16.7	1013.4	736
**2024-06-16**	23.9	15.9	30.2	0	7.4	20.4	1011.4	566
**2024-06-17**	24.3	19.2	30.4	4.3	5.9	14.8	1015.2	617
**2024-06-18**	25.9	18.5	33	0	14.7	16.7	1017.2	713
**2024-06-19**	27.4	19.1	36.4	0	7.2	18.5	1015.5	738
**2024-06-20**	28.2	21.3	34.4	0	6.5	18.5	1017.4	683
**2024-06-21**	29.3	21	36.6	0	7.2	20.4	1015.9	699
**2024-06-22**	28.7	22.5	37.4	0	10.8	43	1009	537
**2024-06-23**	25	19.6	31.1	1.3	11.2	29.6	1009.9	317
**2024-06-24**	25.8	19.2	32.7	0	12.7	27.8	1013	519
**2024-06-25**	26.5	19.1	32.5	0	7.4	22.2	1014.2	426
**2024-06-26**	24.1	19.6	29.9	0	16.2	47	1012.7	387
**2024-06-27**	21.7	16.3	27	1	8.1	22.2	1011.1	218
**2024-06-28**	24.6	17.3	30.7	1.8	5.9	14.8	1014.4	456
**2024-06-29**	27.6	19.1	34.2	0	6.6	14.8	1017	685
**2024-06-30**	28.5	21.4	35.8	0	11.9	29.6	1012.1	689
**2024-07-01**	27.3	21.6	33.3	0	11.5	43	1009.4	619
**2024-07-02**	20.1	17.6	22.5	26.2	12.2	54	1007.9	20
**2024-07-03**	19.5	12.7	25.3	1	8.2	24.1	1008	505

Note: Date format: YYYY-MM-DD; T_{avg}–average air temperature (°C); T_{min}–minimum air temperature (°C); T_{max}–maximum air temperature (°C); Prcp–precipitation/rainfall (mm); W_{spd}–average wind speed (km/h); W_{pgt}–peak wind gust (km/h); Pres–average air pressure (hPa); T_{sun}–sunshine duration (min).

https://doi.org/10.3390/jox16030102

Table S3. Element-specific AAS conditions and validated method-performance parameters for heavy metal determination.

Element	AAS mode	Wavelength (nm)	Calib. range	MDL (mg/kg)	PQL (mg/kg)	Linear range	Accuracy (%)	Precision (RSD, %)
**Cr**	FAAS	357.9	0.05–1.5	1.11	2.25	≤ 2.50	99.6	4.37
	GFAAS		2.5–25	0.022	0.045	≤ 25.25	99.56	3.00
**Ni**	FAAS	232.0	0.25–2.0	9.3	19	≤ 2.00	101.0	7.86
	GFAAS		10–100	0.053	0.107	≤ 101	98.17	4.00
**Cu**	FAAS	324.8	0.1–2.5	1.06	2.15	≤ 2.50	106.5	7.37
	GFAAS		2.5–50	0.022	0.045	≤ 50	99.44	3.01
**Zn**	FAAS	213.9	0.05–1.5	0.57	1.2	≤ 1.50	86.3	6.54
	GFAAS		/	/	/	/	/	/
**As**	FAAS	193.7	/	/	/	/	/	/
	GFAAS		5–100	0.064	0.130	≤ 99	98.52	5.54
**Cd**	FAAS	228.8	0.05–1.5	0.71	1.45	≤ 1.00	98.2	6.87
	GFAAS		0.5–10	0.0076	0.015	≤ 10	99.12	2.55
**Pb**	FAAS	217.0	0.6–10	12.5	25	≤ 10.00	103.9	9.68
	GFAAS		10–100	0.146	0.3	≤ 100	98.03	4.03

Note: FAAS–flame atomic absorption spectrometry; GFAAS–graphite furnace atomic absorption spectrometry; Calib. range–calibration range; MDL–method detection limit; PQL–practical quantification limit; RSD–relative standard deviation. FAAS and GFAAS measurements followed EPA 7000B and EPA 7010, respectively. Calibration and linear ranges are expressed in mg/L for FAAS and in μg/L for GFAAS. Method-performance parameters refer to the validated sediment/soil/sludge analytical method.

References

1. Meteostat Historical Weather and Climate Data Available online: https://meteostat.net/ (accessed on 18 May 2026).
